# Threshold fertility for the avoidance of extinction under critical conditions

**DOI:** 10.1371/journal.pone.0322174

**Published:** 2025-04-30

**Authors:** Diane Carmeliza N. Cuaresma, Hiromu Ito, Hiroaki Arima, Jin Yoshimura, Satoru Morita, Takuya Okabe

**Affiliations:** 1 Graduate School of Science and Technology, Shizuoka University, Shizuoka, Japan; 2 Institute of Mathematical Sciences and Physics, College of Arts and Sciences, University of the Philippines Los Baños, Laguna, Philippines; 3 Department of International Health and Medical Anthropology, Institute of Tropical Medicine, Nagasaki University, Nagasaki, Japan; 4 Department of Biological Sciences, Tokyo Metropolitan University, Tokyo, Japan; 5 Marine Biosystems Research Center, Chiba University, Chiba, Japan; 6 Graduate School of Integrated Science and Technology, Shizuoka University, Shizuoka, Japan; National Cheng Kung University, TAIWAN

## Abstract

The developed countries now face a low fertility crisis. The replacement level fertility (RLF) is conventionally considered to be 2.1 children per woman, in which demographic stochasticity arising from random variations in individual offspring numbers is ignored. However, the importance of demographic stochasticity casts doubts on the adequacy of the replacement level fertility of 2.1, especially in a small population. Here, we investigate the extinction threshold for the fertility rate of a sexually reproducing population caused by demographic stochasticity. The results indicate that the fertility rate should exceed 2.7 to avoid extinction. The extinction threshold is reduced by a female-biased sex ratio. We argue that the present results explain the observed phenomena of female-biased births under severe conditions as an effective way to avoid extinction. Furthermore, since fertility rates are below this threshold in developed countries, family lineages of almost all individuals are destined to go extinct eventually.

## Introduction

The underpopulation crisis has been a serious threat to the sustainability of developed countries. The worldwide total fertility rate (TFR, the number of children per woman) has dropped from 5.3 in the 1960s to 2.3 in 2023 [1,2]. At present, two-thirds of the world’s population lives in areas where the TFR is below the replacement level fertility (RLF) [1,3–6]. The replacement level fertility is the TFR at which the population replaces itself from generation to generation without any population growth [7]. While the RLF is considered 2.1 children per woman in developed countries with low mortality [5,7], modernization and development have decreased the TFR due to the increased opportunity cost of having children [8,9]. Thus, developed countries are seeing very low TFRs. For example, all Group of Seven (G7) countries have fertility rates below RLF: Italy at 1.29, Japan at 1.30, Canada at 1.47, Germany at 1.53, the United Kingdom at 1.57, the United States of America at 1.66 and France at 1.79 [1]. Europe’s and some developed Asian countries’ TFRs have persistently been below the RLF since the 1980s [1], despite their efforts and policies to encourage having children [9–11]. Especially, the Republic of Korea has the lowest fertility rate of 0.87 in 2023, and TFR below the RLF since 1998 [1,11]. Japan’s population is projected to decline by 31% in every generation if fertility rates remain below the RLF [9], threatening future generations.

Conventionally, the RLF assumes low mortality rates and a nearly 1:1 sex ratio [4,6,12]. Moreover, it has been implicitly assumed that the population size is so large that the law of large numbers holds. However, it has been well-acknowledged in ecological studies that stochasticity arising from random variations in individual numbers, called demographic stochasticity, may play an important role in the extinction problem that needs to be taken seriously [13–18]. Especially, small populations are significantly affected by demographic stochasticity, which may cause extinction by unpredictable, random processes. Thus, we have a good reason to question the adequacy of the conventional RLF as a general index for the sustainability of a shrinking population. Moreover, previous studies have challenged the conventional RLF, suggesting that each country’s RLF should depend on its own sex ratio at birth and mortality rates [5]. Skewed sex ratios result in the so-called “marriage squeeze,” which leaves some adults without partners and childless [19–21]. The mortality of individuals below reproductive age influences how many individuals survive to reproduce. Studies have found that an RLF of 3.3 is required in areas where the survival rate drops to 0.60, like Afghanistan, Burundi, and Sierra Leone [12,22]. Thus, there needs to be a reliable population model that should reflect the locality’s mortality level and sex ratio at birth and a model for stochastic events [5,16].

In asexual (unisexual) reproduction models, it has been well known that the threshold growth rate for extinction becomes larger than unity under demographic stochasticity [23–25]. We here explore the extinction threshold in a sexual reproduction model. In this study, we evaluate the extinction probability *P* , i.e., the probability for a lineage of a single adult female to go extinct eventually, based on a branching process model. A branching process is a stochastic process used to describe the dynamical variation of population growth [26–29], in which each individual in the present generation produces some random number of individuals in the next generation. It is assumed that the average number of offspring, *b*, does not depend on the size of the population, i.e., there is no density dependence. We consider a stochastic population where each female gives birth to a Poisson distributed number of offspring, among which the numbers of males and females are binomially distributed with the ratio r:1−r. Furthermore, we consider male- and female-specific mortality rates, $m_{m}$ and $m_{f}$, to see the possible effect on the survival of the population.

## Materials and methods

We consider a sexually reproducing population of non-overlapping generations. Each female gives birth to a random number of offspring if there are males in the population. Specifically, we introduce four parameters based on the following assumptions: (i) The number of offspring follows the Poisson distribution with mean *b*, the fertility or birth rate. The Poisson distribution is a probability distribution that expresses the probability to have a whole number. With mean *b*, the probability or the ratio of females giving birth to *k* offspring is given by $b^{k}e^{-b}/k!,$ where *e* is the base of the natural logarithm and k! is the factorial of *k*. The offspring number *k* takes one of whole numbers, 0, 1, 2, 3, etc., according to the probability distribution. Thus, each female has her number of offspring that varies from zero to any big number, although the probability to have a big number is exponentially negligible. For mean fertility b=2, for instance, a total of 68% (95%) of females has less than three (five) offspring, among which 14% has no offspring. (ii) Male and female offspring are binomially distributed with the male ratio *r* (the female ratio 1−r). In the binomial distribution, the probability that a total of *k* offspring consists of *l* males and k−l females is given by $cr^{l}{\left(1-r\right)}^{k-l}$, where $c=k!/(l!/\left(k-l\right)!)$ is a binomial coefficient and *l* is a whole number from 0 to *k*. For example, for k=2 and r=1/2, sex composition of two offspring turns out to be male: female=2: 0, 1: 1 and 0: 2 with probability 25%, 50% and 25% because c= 1, 2 and 1, respectively. In what follows, *r* may be called the sex ratio at birth in accordance with the prior study [30]. The binomial distribution follows if childbirth events are independent from each other, so that this assumption is quite realistic. (iii) Male and female offspring die before reproduction, with the probability $m_{m}$ and $m_{f}$ (male- and female-specific mortality), respectively. As a matter of fact, this is more of a definition of symbols than an assumption, for their numerical values depend on the reproductive age or generation years. Sex-specific mortality is introduced not only to see how it modifies the results but also for the sake of comparison with the previous study [30]. In this respect, the present interest lies not in its quantitative impact but in its qualitative effect.

We are interested in the extinction probability that a lineage starting from a single female goes extinct. We may trace descendants of a pair of a female and a male. The number as well as the male: female ratio of the next generation (generation 1) are determined randomly according to the probability distribution as specified. Similarly, the sex composition of generation 2 is obtained as outcomes of random events, and so forth. The descendants proliferate if chance favors. However, this is not always the case. The lineage terminates when some generation happens to have no male or female. This probability of extinction, denoted as *P*, may be evaluated numerically as the ratio of the cases where the lineage goes extinct by repeating the history of reproduction a large number of times, while it is also obtained by analytical method of solving a recurrence relation (see Supporting information for details). Numerical simulations are performed with Python (see Data and materials availability statement). This study did not make use of human or animal subjects and/or tissue.

## Results

**Fig 1:**
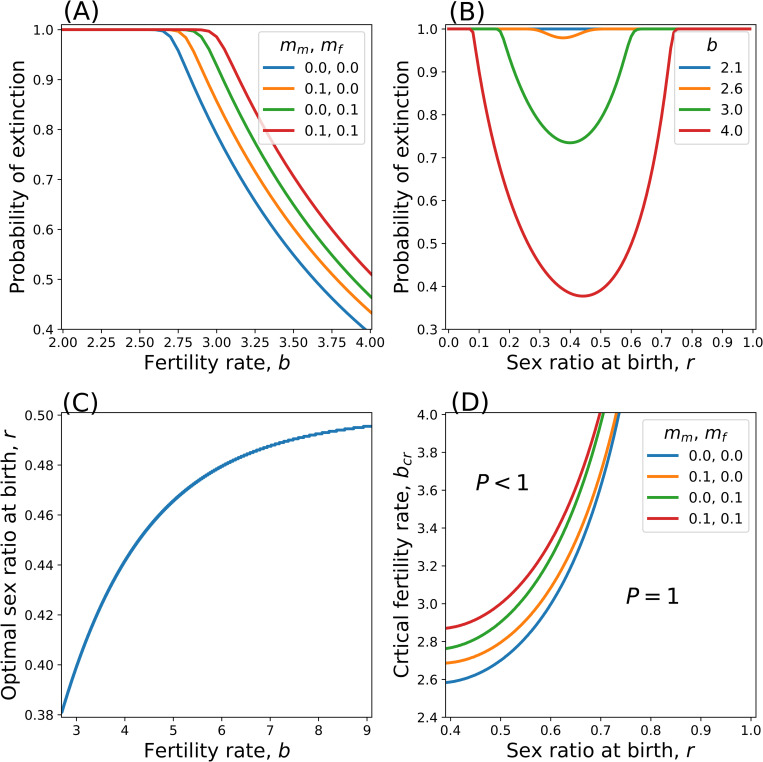
The extinction probability *P* and the critical fertility $b_{cr}$. **(A)** *P* versus fertility *b* (r=0.5), **(B)** *P* versus sex ratio *r* ($m_{m}=m_{f}=0$), **(C**) Optimal sex ratio *r* versus fertility *b*, and **(D)** $b_{cr}$ versus *r* for $\left(m_{m},m_{f}\right)=\left(0,0\right),\left(0.1,0\right),\left(0,0.1\right)$ and. $\left(0.1,0.1\right).$

Fig 1 shows the way the extinction probability *P* varies against the fertility rate b, sex ratio r, and mortality rates $m_{m},m_{f}$. The results in panels (A)-(C) indicate that a female-biased sex ratio mitigates the extinction probability. Fig 1(D) shows the sex-ratio dependence of the critical fertility rate $b_{cr}$, which is the fertility rate below which the extinction probability becomes unity, i.e., P=1 for $b\leq b_{cr}$ while P<1 for $b>b_{cr}$. For r=0.5 and $m_{m}=m_{f}=0$, the critical fertility rate is approximately 2.7, significantly higher than the conventional RLF. The critical fertility decreases as *r* decreases.

**Fig 2:**
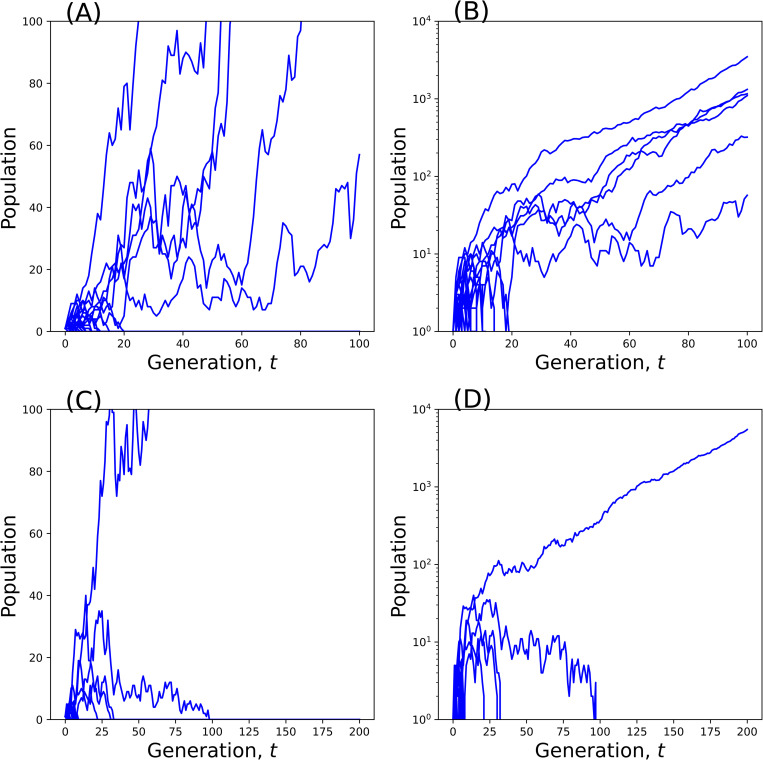
The population with subcritical fertility goes extinct except for a few exceptions that keep growing. **(A,** **B)** r=0.5,b=2.1, **(C,** **D)** r=0.5,b=2.05. Among 100 independent replicates, only six runs in (A, B) and one run in (C, D) survive to grow. ($m_{m}=m_{f}=0$, 100 trajectories overlapped).

Fig 2 shows the dynamics of simulated populations below the critical fertility rate $b_{cr}$. Some exceptional populations may persist despite the 100% extinction probability (P=1). Among 100 independently simulated populations, only a few survive, i.e., six for b=2.1 and one for b=2.05. These exceptional populations keep growing, while all the other populations go extinct within 20 generations without reaching ten individuals.

**Fig 3:**
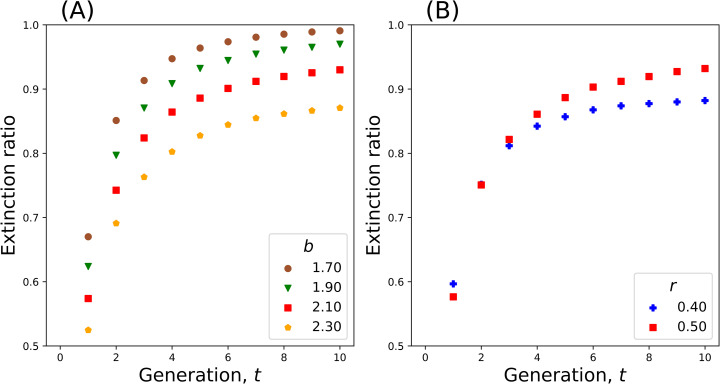
The ratio of extinct populations is plotted against generation number. **(A)** b=1.7,1.9,2.1 and 2.3, and **(B)** r=0.4 and 0.5 for b=2.1. For a subcritical fertility ($b<b_{cr}≃2.7$), most populations do not survive a few generations. ($m_{m}=m_{f}=0$, 10,000 replications).

Fig 3 shows how the ratio of extinct populations approaches 1 (almost sure extinction) as generation *t* increases ($b<b_{cr}$). Low fertility rates b=1.7 and 1.9 show the steepest approach to 1 within ten generations. In contrast, fertility rates closer to $b_{cr}$b=2.1 and 2.3show a more gradual approach. Indeed, the ratio of extinction appears to be significantly less than 1 at generation 10. In Fig 3(B), the ratio of extinct populations is smaller for r=0.4 than for r=0.5. This is consistent with the result that $b_{cr}$ is smaller for r=0.4 than for r=0.5 (Fig 1(D)).

**Fig 4:**
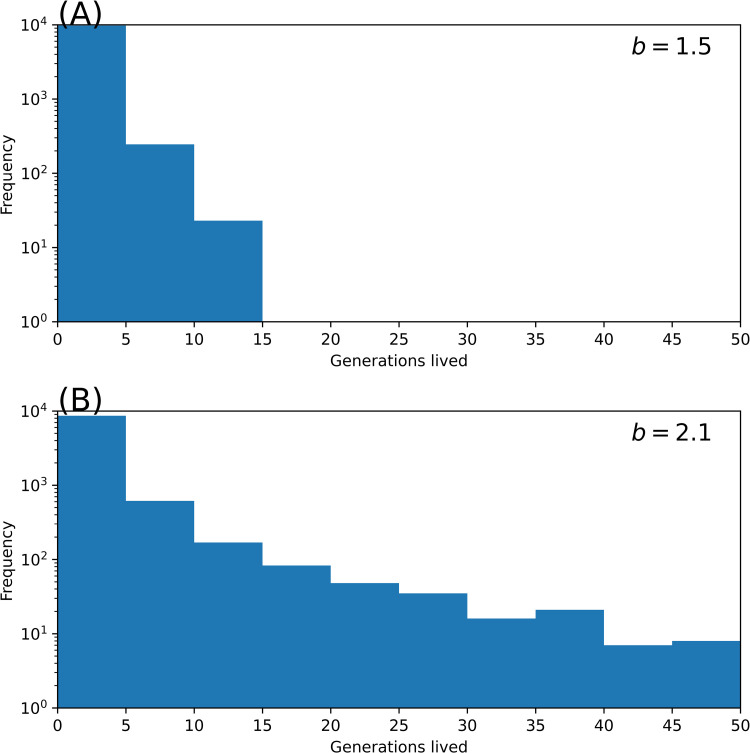
The histogram of survived generations for the population with subcritical fertility. **(A)** b=1.5, and **(B)** b=2.1. ($r=0.5,m_{m}=m_{f}=0$10,000 replications).

In Fig 4, we show the histogram of the survived generations for b=1.5 (Fig 4(A)) and b=2.1 (Fig 4(B)) ($r=0.5,m_{m}=m_{f}=0$). In both cases ($b<b_{cr}$), a vast majority of the population lived for only five generations. Note the logarithmic scale in the *y* axis. As the fertility rate *b* approaches the critical value $\left(b_{cr}≃2.7\right)$, the exceptional populations on the tail of the histogram increase, i.e., the histogram becomes fat tailed. It should be emphasized that the survival of a subcritical population is not impossible but highly improbable.

## Discussion

In the present analysis, we made various assumptions for the sake of tractability and representational convenience. The assumption of no overlapping generations (or discrete generations) is a notable simplification that is not met in many mammals including humans. In fact, overlapping generations are seen in species that reproduce more than one time during their long lifetimes. The most notable effect due to overlapping generations would be found in the speed of evolution accelerated by enhanced genetic diversity. Nonetheless, the time scale of population size variation in the present study is too short to comparable to this evolutionary scale. In this respect, possible variation in generation years should have more impact on the present study. We assumed a constant fertility rate *b*, with which the female population grows per generation, if it were not for the risk of extinction. The rate of growth per generation can happen to fluctuate owing to variations in generation years, which, however, was outside the scope of the present interest. Another assumption is the Poisson distribution for the offspring number. This assumption is more of a convenience than a necessity. We have dealt with the problem of a chance event that a female gives birth to children of the average number *b*. The actual number fluctuates around the mean b and how it fluctuates is specified with a probability distribution function. The Poisson distribution is a distribution characterized with a single parameter, with which mean and variance agree. The important point for us is that the children number can be zero with non-zero probability, i.e., there is always a minority of females who do not have any child. The present analysis focused on the consequence of this minor events that are usually ignored. It is generally expected that the ratio of females to have no child at all should decrease as the mean fertility rate increases. The decrease is exponential in the Poisson distribution (the ratio is 1 for b=0 and 0.14 for b=2). Without specific information on how they vary in real data, we consider it judicious not to make any hypothesis than to adopt a single-parameter description by the Poisson distribution. Thus, this limitation suggests further research once the correlation between the mean fertility rate and the ratio of females with no child is quantified. While a more sophisticated model assumption may cause minor modifications, it is unlikely to have a major impact on the findings and conclusions of this study. Lastly let us remark that the present interest lies in the possible consequences of zero childbirth. We assumed specific values for the mortality rate (0.0 or 0.1) for the sake of presentation. The purpose of it was to show the manner in which mortality and its sex dependence affect the results. The same remark applies to the sex ratio.

The world experienced its peak growth rate in the 1960s [31–33] and the global population has continued to increase and is projected to reach 8.5 billion in 2030 [32]. Thus, studies and policies focused more on the carrying capacity of the human population and curbing population growth [34–38]. However, at present, two-thirds of the world’s population lives in areas where the total fertility rate is below the replacement level fertility (RLF) [1,3–6].

It is considered that replacement level fertility (RLF) of 2.1 children per woman maintains the population size based on explicit or implicit assumptions [4,6,12]. Casting doubt on the implicit assumption of a large (infinite) population size, the present study investigated the extinction problem of a population whose size fluctuates randomly from generation to generation. For the even sex ratio r=0.5 and no mortality $m_{m}=m_{f}=0$, we obtained the critical fertility $b_{cr}≃2.7$, which is considerably larger than 2.1. This means the population goes extinct with certainty even if the RLF is achieved. This may appear paradoxical. However, the significance of the present result should be critically notable in small populations, especially those on the verge of extinction. Under the condition that fertility is below the critical value ($b<b_{cr}$), even if it is above RLF, almost all, if not all, populations do not survive a few generations before going extinct (Fig 2).

It should be remarked that this condition has already been met in developed countries. Extinction is not an immediate issue owing to the large population size in these countries. However, the present results have a profound implication from an individual perspective: The family lineages of almost all individuals are destined to go extinct, whereas very few exceptions may survive for many generations (Figs 3 and 4). Languages also face the risk of extinction, with at least 40% of more than 6,700 spoken languages in the world threatened to disappear within the next 100 years [39,40]. The extinction of a language results in the disappearance of a culture, art, music and oral traditions [39].

Understanding the impact of a biased sex ratio is important to the conservation of small populations on the brink of extinction [41–44]. A skewed sex ratio can have profound implications for the persistence of population [45,46]. According to the present results, the less fertility *b*, the more effective the female bias reduces extinction risk. Hence, we argue that the phenomena of female bias observed under severe conditions are adaptive for the avoidance of extinction. Human parents exposed to various stressors tend to have a female-biased sex ratio for their offspring, while the sex ratio is normally male-biased at 1.057 [1]. Specifically, psychological distress [47–49], non-optimal reproductive/metabolic conditions [50], binge eating and other eating disorders [51], elevated blood pressure and high caloric intake [48], exposure to chemicals (by smoking [52], inhalation of particulate matter [53], and by exposure to insecticides and medical disinfectants [54], dioxin [55] and Methylmercury [56]), socio-economic stress [57] including terrorism [58,59] and economic collapses [60,61], and natural disaster [62,63] have all been reported to have an influence towards a female-biased sex ratio. Moreover, the female-biased sex ratio subjected to stress conditions have also been observed in mammal populations, such as in cattle [64], pigs [65], rats [66], and mice [44]. Thus, the mechanism for adjusting the sex ratio in response to stressful conditions (i.e., via a stress hormone, glucocorticoids [44]) is potentially adaptive for parents’ fitness [67].

While the human population is unlikely to face immediate extinction, our findings provide a framework for extinction avoidance, informing conservation efforts and breeding programs for endangered species [68]. The survival of a population is a dynamic and multi-variable problem. The extinction problem should be a critical issue in small populations of isolated communities, not to mention endangered species [68]. The current study focused on the probability of extinction due to demographic stochasticity. Further study can be done to consider other factors like migration, density dependence and environmental stochasticity.

## Supporting information

S1 AppendixDerivation of $P\left(t\right)$.(DOCX)
