# Supplementary material for: Threshold fertility for the avoidance of extinction under critical conditions
Source: PLoS One. 2025 Apr 30;20(4):e0322174. doi: 10.1371/journal.pone.0322174 (PMC12043152; doi:10.1371/journal.pone.0322174)
Supplement: S1 Appendix — Derivation of Pt. (DOCX) [file pone.0322174.s001.docx]

**Title:** Threshold fertility for the avoidance of extinction under critical conditions

**Authors:** Diane Carmeliza N. Cuaresma^1,2^, Hiromu Ito^3^, Hiroaki Arima^3^, Jin Yoshimura^1,3,4,5,6^, Satoru Morita^1^ and Takuya Okabe^7^*

**Supporting information**

**S1 Appendix.** Derivation of $P(t)$

Let us consider a sexually-reproducing population of non-overlapping generations, which is traced only through females. Each female gives birth to an independent and identically distributed number of offspring, provided that there are males. The initial female, which we refer to as generation 0, has offspring called generation 1. The offspring of generation 1 are the generation 2, and so on. We evaluate the probability $P$ that a lineage starting from a single female goes extinct. For this purpose, we introduce and derive an expression for $P(t)$, which is the probability of extinction of a lineage starting from a single adult female in some generation $t$. By extinction, we mean the event that the population is 0 except for a finite number of generations.

We obtain a recurrence relation for $P(t)$ under the following assumptions:

(i) The number of offspring of each female is a $N$, where $N$ is a realization of a random variable $X$ that follows the Poisson distribution with mean $b$. Here, $b$ is the fertility rate.

(ii) For each female, there are $M$ males among the $N$ offspring. $M$ is a realization of a random variable $Y$ that follows the Binomial distribution with parameter $r$, the sex ratio at birth.

(iii) The male and female offspring die with probability $m_{m}$ and $m_{f}$, respectively, before reaching maturity.

(iv) The probability of extinction of a lineage starting at generation $t$ is equivalent to the extinction of all lineages starting from the offspring of females in generation $t$. Thus, a lineage is at risk of extinction when

(a) all $M$ males die before reaching maturity, which is given by the probability $m_{m}^{M}$, and

(b) $N-M-f$ females die before reaching maturity and the descendants of the $f$ that survived go extinct in the next generation, $\left( \begin{matrix} N-M \\ f \end{matrix} \right)m_{f}^{N-m-f}\left( 1-m_{f} \right)^{f}P\left( t+1 \right)^{f}$.

In addition, we keep track of the size of each generation and do not consider the time offspring are born. We also do not consider family relationships. Note that assumptions (i) to (iv) define a particular case of a branching process.

Now, considering all possible numbers of offspring, the probability that the lineage of a single adult female in generation $t$ goes extinct is given by

$P\left( t \right)=\sum_{N=0}^{\infty} \left( \sum_{M=0}^{N} \frac{b^{N}}{N!}e^{-b}\left( \begin{matrix} N \\ M \end{matrix} \right)r^{M}\left( 1-r \right)^{N-M}\left( m_{m}^{M}+\left( 1-m_{m}^{M} \right)\sum_{f=0}^{N-M} \left( \begin{matrix} N-M \\ f \end{matrix} \right)m_{f}^{N-M-f}\left( 1-m_{f} \right)^{f}P\left( t+1 \right)^{f} \right) \right)$.

By multiplying $e^{b}$ to both sides of the equation and by some algebraic manipulations, we arrive at

| $P\left( t \right)=e^{-br\left( 1-m_{m} \right)}+e^{b\left( 1-r \right)\left\{ (1-m_{f})P(t+1)+m_{f} \right\}}\left( e^{-b\left( 1-r \right)}-e^{-b\left( 1-m_{m}r \right)} \right)$. | Eq. (1) |
| --- | --- |

From Eq. (1), the critical fertility rate $b_{cr}$ (to make $P<1$ for $b>b_{cr}$) is obtained as a solution of

| $1=b_{cr}\left( 1-r \right)\left( 1-m_{f} \right)\left( 1-e^{-b_{cr}r\left( 1-m_{m} \right)} \right)$. | Eq. (2) |
| --- | --- |

Note that $P\left( \infty\right)=0$, so that for sufficiently large $T$, $P\left( T \right)=0$. Then, we can solve Eq. (1) backward by obtaining $P(T-1)$ using $P\left( T \right)=0$, $P\left( T-2 \right)$ using the value of $P(T-1)$, and so on, until we reach $P=P(0)$.
